# Alcohol as a Beverage

**Published:** 1898-02

**Authors:** 


### Alcohol as a Beverage.

This question becomes more and more a medical one in Europe every year, especially in beer and wine-producing provinces. In France it has become a national topic, in which societies, both medical and laymen, and even the government itself, have taken part. The decrease of the birth-rate and increase of insanity, criminality and pauperism in France have been proven to be the result largely of the use of alcohol. This has been ascertained by statistics compiled by the Commissioner of Agriculture under the government, and can not be disputed as the work of sentimentalists. In the Rhine provinces the same alarming statistics have been gathered, and even in England private studies of beer-drinkers and others who use wine regularly add strong confirmation to the alarm felt in France. This alarm is spreading rapidly, not by reformers and temperance efforts, but by well-considered medical studies, and calm, dispassionate papers.

The first question which seems to attract attention is, what are the physiological effects of alcohol on the body? Within six months thirty papers on this topic have appeared in France, and as many more in Germany. In England the number has passed over fifty, and is increasing every month. Of course, a large proportion of these papers are simply repetitions of a few authors, with some additions and opinions of others.

The leading facts in the French papers are that all distilled spirits are paralyzing and narcotic in their action, hence are directly and indirectly poisonous. While wines are in some conditions harmless, they should never be used by young, healthy persons, and can only be taken by the old and enfeebled with safety. These papers indicate the dangers from complex alcohols found in old wines and spirits, and demand that the sale shall be prohibited, and that these alcohols shall be classed as poisons.

The German papers take strong ground against all alcohols as beverages, and condemn both beer and wine except to the aged and infirm. They also show from experiment that alcohols are depressants and paralyzants, and the supposed good effects are due altogether to their anesthetic properties.

The English papers reflect these views, in addition to some new studies of drunkards and excessive drinking, in which the palsied condition is demonstrated by actual physiological measurement.

Some of the great laboratories of Europe, especially in France and Germany, have taken up this subject, and the question of how alcohol affects the organism is asked.

In this country exact studies of alcohol have been taken up by the Committee of Fifty organized to carry on original work in this field. So far they have published a few papers, one "On Alcohol on Digestion," another "On Alcohol on Growth," and one "On Statistics and History of Alcohol." These have brought out in an indirect way the same fact of the anesthetic action of spirits. Several very clear papers by physicians have taken strong ground against all use of spirits, based on clear, sound reasoning. A great variety of facts have been gathered by individuals confirming the same view.

While all this agitation is going on in Europe, very advanced work has been done in this country concerning the inebriate and his malady. The Association for the Study and Cure of Inebriety was formed in 1870, and has been the pioneer of the world in this field. Its researches, showing that inebriety was a symptom, have been accepted all over the world, and credited to this country. It seems to be accepted that alcohol can not be used as a beverage in this country with safety. In nearly all efforts of this kind most disastrous results have followed. Physicians and observers all agree that very few persons are able to use spirits as a beverage long, owing to the peculiar feverish life and living of Americans. Already the excitement of Europe in relation to the use of alcohol is being felt here, and medical men realize that the alcoholic question is not confined to reformers and idealists, but there is a medical and scientific side which calls for the highest judgment and research. In the meantime the manufacturers and dealers of spirits realize that a great revolution is coming up, which perils their future. To the medical man a new field is open for observation, and the question, is alcohol in any form ever a beverage which can be used safely? must be answered by an appeal to facts which have but one meaning.

